# Male Pomegranate (*Punica granatum* L.) Flowers Are Associated with Resource Limitation and Lack Female Reproductive Function

**DOI:** 10.3390/plants15213418

**Published:** 2026-11-03

**Authors:** Reyilamu Maimaitituerxun, Qingli Liang, Mierkamili Maimaiti, Aysajan Abdusalam

**Affiliations:** 1 College of Life and Geographic Sciences, Kashi University, Kashi 844000, China; 2 Key Laboratory of Biological Resources and Ecology of Pamirs Plateau in Xinjiang Uygur Autonomous Region, Kashi University, Kashi 844000, China

**Keywords:** *Punica granatum* L., sex allocation, resource allocation, pollination biology, fruit tree

## Abstract

Sexual system plasticity strongly shapes plant reproductive fitness. However, empirical studies on andromonoecy have mostly focused on wild species, leaving gaps in our knowledge regarding cultivated fruit trees. In this study, we aimed to investigate the sexual system characteristics and adaptive responses to resource conditions of pomegranate (*Punica granatum* L.) in Kashi, Xinjiang, China. We assessed floral morphology and biomass, conducted resource manipulation treatments, pollen viability assays, and controlled pollination experiments, and observed pollinators. Our results indicated functional andromonoecy in pomegranate, in which male flowers originate from pistil degeneration, potentially in response to resource limitations within inflorescences. Hermaphroditic flowers showed advantages in morphology, biomass, floral rewards, and pollinator visitation. Male flowers could not produce fruits as maternal parents but generated viable pollen capable of siring progeny. However, they secreted less nectar and received significantly fewer pollinator visits than hermaphroditic flowers. These findings are consistent with the optimal resource reallocation hypothesis for maintaining andromonoecy, suggesting that male flowers may constitute a resource-saving strategy. Whether the male-flower proportion can serve as a reliable indicator of plant resource status requires further validation.

## 1. Introduction

Sexual systems are crucial determinants of genetic variation and reproductive success, influencing evolutionary processes within populations [1,2,3,4,5,6]. Flowering plants exhibit remarkable diversity in their sexual systems at the individual, population, and species levels. This diversity in plant sexual systems has long been recognized and remains a major focus of research in reproductive biology [2,7]. The evolution from a sex-associated monomorphic system (hermaphroditism) to sex-separated dimorphic systems (dioecy, gynodioecy, and androdioecy) occurs through three pathways. The “direct” pathway involves an immediate transition from hermaphroditism to dioecy via a single mutation. The “dimorphic” pathway passes through intermediate stages with distinct male and female morphs (e.g., gynodioecy or androdioecy). The “monomorphic” pathway involves transient monoecious or hermaphroditic stages before the establishment of separate sexes [8,9,10]. These pathways have led to the evolution of sexually dimorphic systems in angiosperms through repeated independent evolutionary processes (up to 5000 times), which have also resulted in the formation of intermediate transitional sexual systems (monoecism, andromonoecism, and gynomonoecism) [2,5,6,10,11,12]. The existence of these intermediate transitional systems, which are considered evolutionary precursors to dioecy (~6% of angiosperms) [13], is a crucial issue in the evolution of plant sexual systems because the manifestation of sexual plasticity in plant species of this type is regulated by diverse abiotic environmental factors, such as water, temperature, light, and nutrition, and biotic (genetic) regulators [11,12,14,15]. The considerable variation and evolutionary instability of sexual systems in angiosperms directly affect their diversity [2,14,16,17]. Various biotic (including genetic variation, individual size, phenology, and other plant characteristics) and abiotic (including extreme temperatures, availability of water, light, nutrients, and CO_2_ levels) factors influence sexual systems [2,11,18,19]; environmental stress can bias sex ratios and modify sexual expression patterns. Thus, sexual expression and plasticity can affect the evolutionary processes of individual plants, populations, and species, leading to diversity in sexual systems within species.

Andromonoecy is a transitional phase in the evolutionary trajectory from hermaphroditism to dioecy [10]. This sexual polymorphism occurs across multiple angiosperm families but is relatively rare at the species level. Andromonoecy may have originated from the degeneration of pistils in hermaphroditic flowers [9,20], production of additional male flowers by hermaphroditic ancestors, or development of hermaphroditic flowers from female flowers of monoecious plants [21]. These adaptations may enable plants to evade self-pollination and facilitate cross-fertilization [22]. Multiple hypotheses have been proposed to explain their evolutionary significance, including resource reallocation [23], pollen donation [24], attraction [25,26], reproductive assurance [9,21], and sexual interference [27,28,29]. The resource reallocation hypothesis posits that male flowers incur lower costs than hermaphroditic flowers, leading to more efficient resource allocation [30,31]. Furthermore, male flowers do not affect female fitness; instead, they compensate for the resources invested in male flower production and enhance male fitness [20,32]. The pollen donation and attraction hypotheses propose that male flowers generate a greater quantity or higher quality of pollen and are more successful in pollen export than hermaphroditic flowers. Male flowers produce pollen that can be transferred to other plants via pollinators, thereby enhancing male reproductive success [20,33,34,35]. Furthermore, male flowers exhibit conspicuous floral displays that facilitate pollinator attraction, visitation frequency, and female fitness [20,36]. In 1982, Bertin suggested that the optimal allocation hypothesis offers the most satisfactory explanation for the evolution and maintenance of andromonoecy [23]. More recently, although researchers have advocated for the optimal resource allocation hypothesis for wild plants [30], such evidence is scarce for cultivated plants (e.g., fruit trees) [37].

Pomegranate (*Punica granatum* L.), belonging to the family Lythraceae, is a deciduous shrub or small tree and the only cultivated species in this family. This species is found in approximately 30 countries but is predominantly distributed across Central Asian regions characterized by subtropical and temperate climates, such as Iran, Afghanistan, and the Caucasus. Pomegranate has been cultivated for more than 2000 years in northern and southern China, with its distribution spanning eight major production areas: Shandong, Henan, Anhui, Jiangsu, Shaanxi, Sichuan, Yunnan, and Xinjiang [38]. This ancient fruit tree is recognized for its medicinal and nutritional attributes [39]. To date, studies on *P. granatum* have primarily focused on cracking and sunburn damage, genome analysis and comparison, irrigation techniques, biochemical characterization of various parts of the tree, genetic diversity and germplasm resource evaluation, and standardization of cultivation and agronomic techniques [40,41,42,43,44,45,46,47].

Based on floral characteristics, some researchers classify pomegranate flowers into normal (fertile hermaphrodite) and degenerate (infertile male) groups [48,49], whereas others classify them as infertile (male flower), fertile (hermaphrodite), and intermediate-type flowers [50]. The sexual system of pomegranate has long been recognized as functional andromonoecy. Early horticultural work documented this condition [51], and subsequent microscopic studies have characterized the two floral morphs in detail, showing that male flowers bear shortened pistils of variable height with rudimentary, degenerating ovules and styles in which pollen tubes are rarely observed, whereas hermaphroditic flowers have a discoid, exudate-covered stigma, elongate style, and numerous anatropous ovules [52]. The proportions of the two flower types are not fixed; they vary strongly among cultivars and genotypes, seasons and flowering waves, and flowers of different positions and sizes within an inflorescence [53,54,55,56,57]. However, previous studies were largely descriptive or cultivar-focused; the quantitative dynamics of sex expression across inflorescence sizes and flowering waves, role of resource availability in regulating floral sex determination, and functional consequences of the two floral morphs for pollinator attraction and pollen donation remain poorly understood. Notably, intermediate-type flowers were not investigated in the present study, and we focused only on male and hermaphroditic flowers.

Owing to the substantial variation among pomegranate varieties, there is currently no unified classification for their flowers. Therefore, in the present study, we investigated the pollination biology of pomegranate orchards in Kashi City, Xinjiang, China. Individual plants simultaneously produced flowers of two different shapes, of which only one produced fruit. Initially, we speculated that this species had an andromonoecious sexual system. We characterized its sexual system and sexual expression processes to answer the following questions: (1) What is the sexual system of this species, and what are the characteristics of the sexual system at the floral, inflorescence, individual, and population levels? (2) If this species exhibits an andromonoecious sexual system, how do male flowers affect pollination and reproduction? (3) What are the reasons for the emergence of male flowers? This study elucidates the characteristics of the sexual system of this species and provides a theoretical basis for further research on progeny generation, fruit yield, and quality improvement.

## 2. Results

### 2.1. Variable Floral Morphology, Biomass Allocation, and Resource Investment in Two Types of Flowers

**Figure 1:**
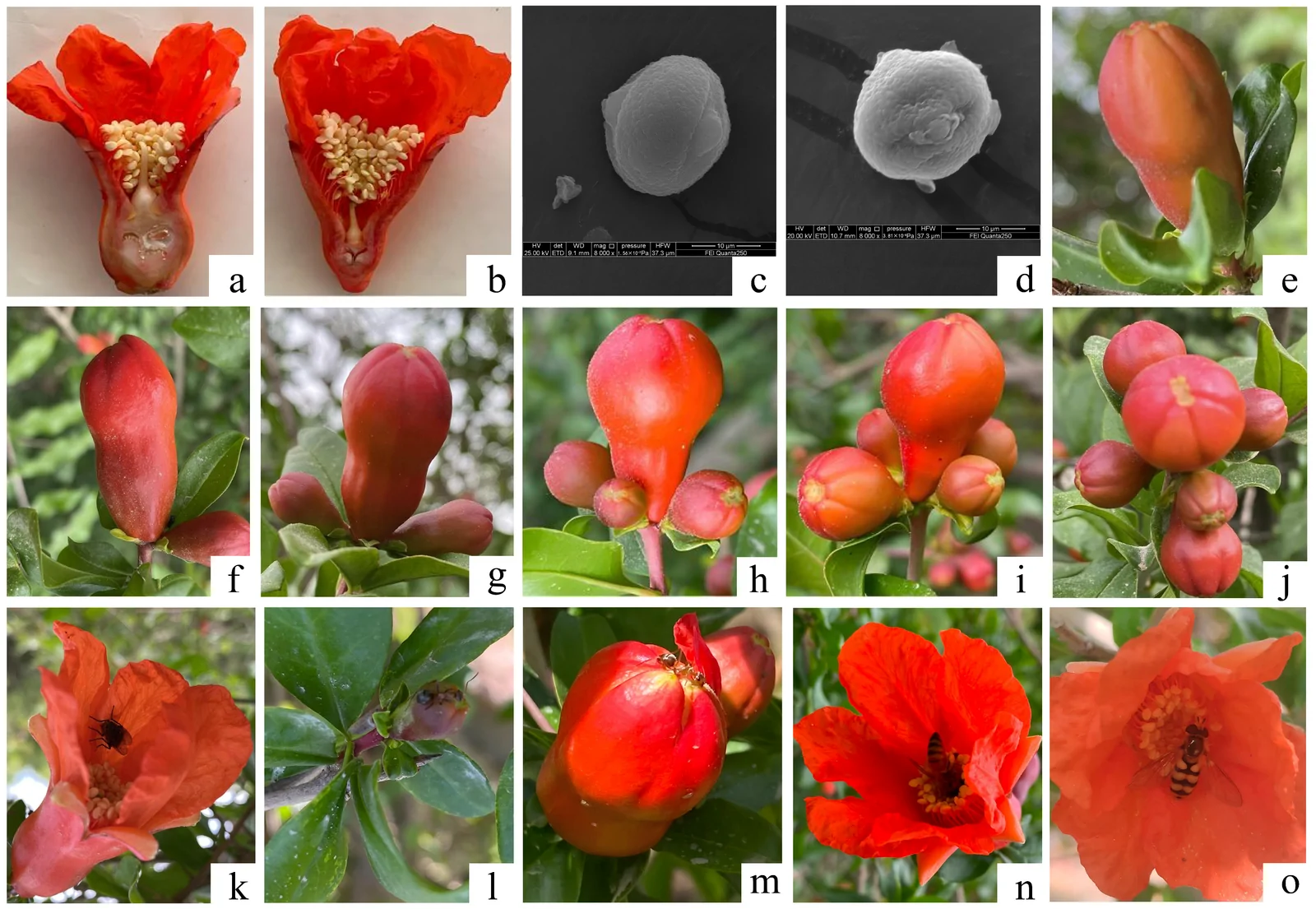
Longitudinal sections of *Punica granatum* L. flowers of (**a**,**b**) different sexes. (**a**) Flowers with normal pistil development, (**b**) largely degenerated pistils. Pollen grains of flowers with (**c**) normal pistil development and (**d**) largely degenerated pistils. (**e**–**j**) Inflorescences of different sizes. (**k**–**o**) Floral visitors of *Punica granatum* L. (**k**): Syrphid fly sp., (**l**,**m**): *Tetramorium* sp., (**n**): *Apis mellifera*, (**o**): *Ischiodon scutellaris* (Fabricius).

Based on pistil development, *P. granatum* flowers were categorized into two types: those with normally developed pistils and those with partially or completely degenerated pistils. Flowers with normal pistils exhibited a vase-shaped calyx tube, a prominently swollen ovary base, 6.11 ± 0.2 stamen whorls, and a style significantly longer than the stamens. Flowers exhibiting pistil degeneration comprised a tubular or bell-shaped calyx, a slightly inflated or narrow ovary base, and 5.7 ± 0.3 stamen whorls. The style was a similar length to or slightly shorter than the stamens (Figure 1).

Morphometric comparisons showed that the flowers with normal pistil exhibited significantly greater values than the pistil-degenerated flowers across all analyzed organ traits (corolla tube length, corolla diameter, calyx length, petal width, anther length, stamen distribution range, style lengths, ovary dimensions, stamen basal position, and pollen surface area; *p* ≤ 0.05 to ≤0.001; see Table 1), whereas corolla tube width, calyx width, petal length, stamen length, anther width, and filament length did not significantly differ (*p* ≥ 0.05). Therefore, although the divergence between floral types extended to several traits, the differences in female organ traits (style length and ovary dimensions), together with the functional sterility of degenerated pistils, constituted the key diagnostic features distinguishing the two floral morphs. These morphological differences, together with the presence of two distinct floral morphs within the same individual, support the interpretation of a typical andromonoecious sexual system [58,59] characterized by hermaphroditic (normal pistil) and male (pistil-degenerated) flowers. Functional validation is provided in Section 2.6.

**Figure 2:**
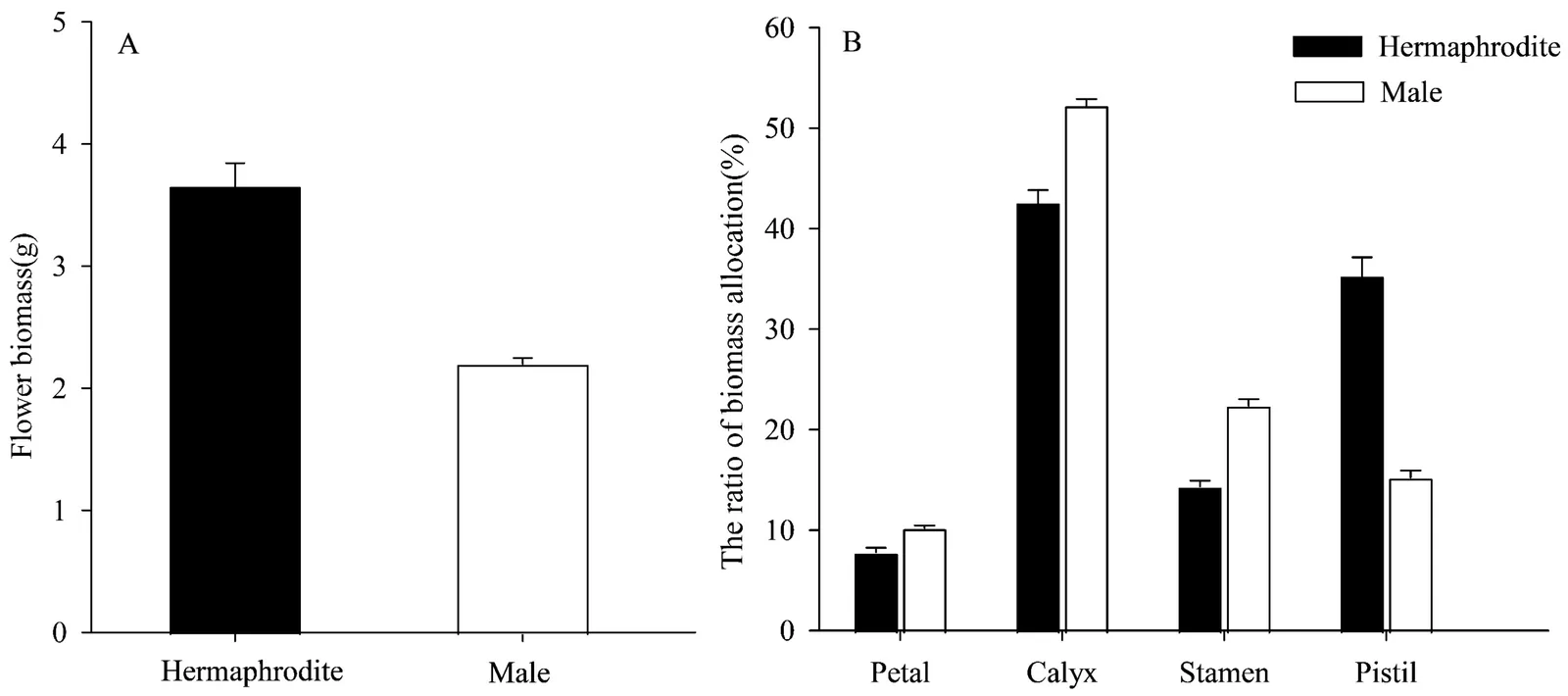
(**A**) Comparisons of total flower biomass and (**B**) proportional biomass allocation to floral organs between hermaphroditic and male flowers of *Punica granatum* L. (mean ± SE, *n* = 15 for each flower morph).

The total flower biomass (fresh weight) also differed significantly between flower types (Wald χ^2^ = 95.05, *p* ≤ 0.001). Hermaphroditic flowers were heavier than male flowers (Figure 2A). Furthermore, male flowers allocated a greater proportion of biomass to the petals, calyxes, and stamens, whereas hermaphroditic flowers invested more in the pistils. The proportional biomass allocation to petals (Wald χ^2^ = 29.25, *p* ≤ 0.001), calyces (Wald χ^2^ = 48.99, *p* ≤ 0.001), stamens (Wald χ^2^ = 70.51, *p* ≤ 0.001), and pistils (Wald χ^2^ = 157.99, *p* ≤ 0.001) varied significantly between flower types (Figure 2B).

### 2.2. Sexual System Characteristics and Floral Sexual Expression

Flower longevity differed significantly between flower types (Wald χ^2^ = 30.19, *p* ≤ 0.001), with hermaphroditic flowers lasting longer (5.6 ± 0.2 d) than male flowers (4.6 ± 0.1 d). Anthesis occurred at 18:00 on the day following calyx cracking, with anther dehiscence commencing the following day for both flower types.

**Figure 3:**
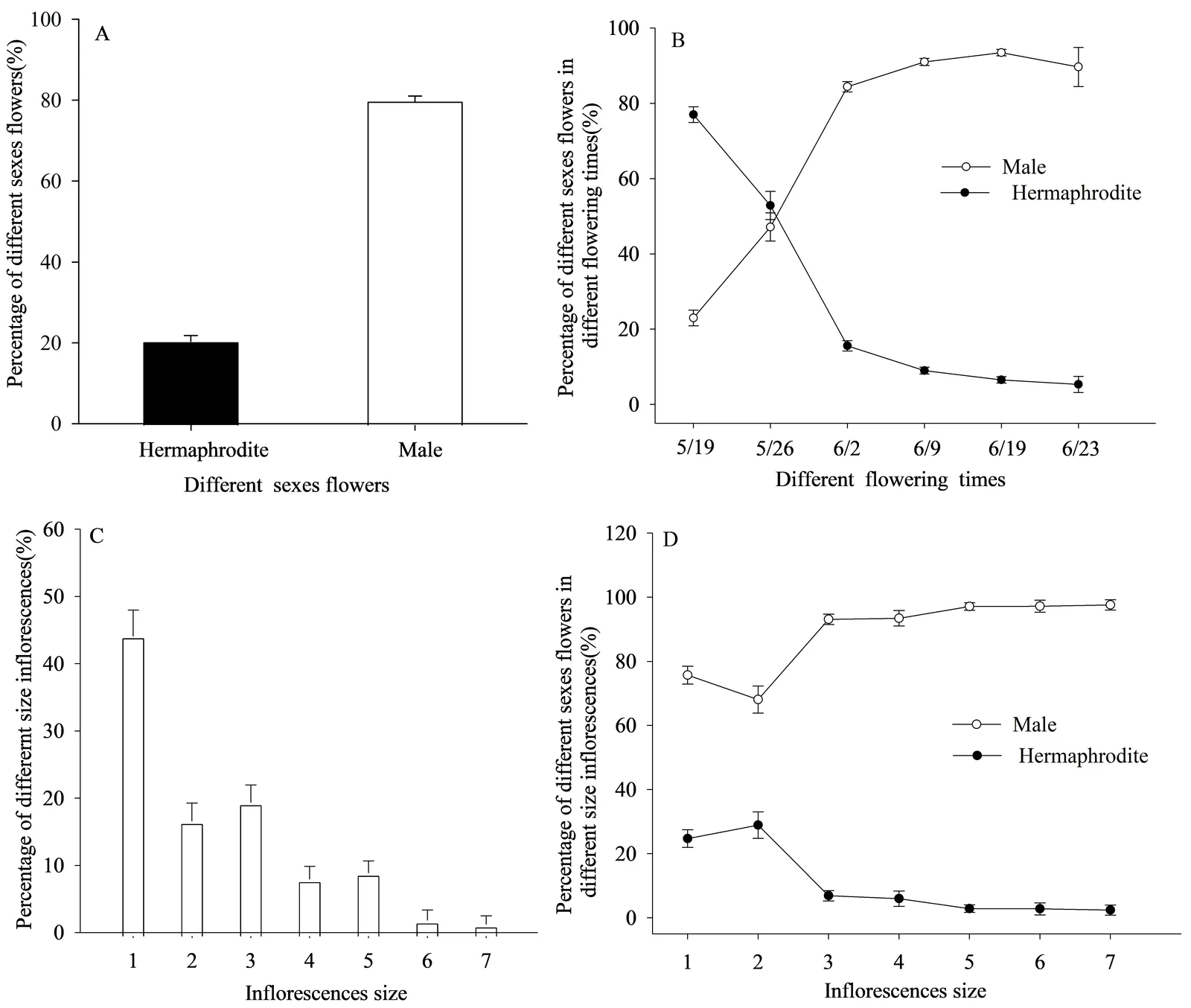
Percentage of *Punica granatum* L.flowers of different sexes on individual plants (**A**) overall and (**B**) at different flowering times in the flowering seson; (**C**) percentage of inflorescences of different sizes and (**D**) flowers of different sexes in inflorescences of different sizes (mean ± SE, *n* = 20 flowers per flower-sex morph).

At the individual level, male flowers dominated the floral composition (79.61 ± 1.43%), significantly outnumbering hermaphroditic flowers (20.39 ± 1.43%) (Wald χ^2^ = 904.78, *p* ≤ 0.001; Figure 3A). Their proportions shifted dynamically across flowering stages (hermaphroditic: Wald χ^2^ = 1080.52, *p* ≤ 0.001; male: Wald χ^2^ = 560.26, *p* ≤ 0.001; Figure 3B). Hermaphroditic flowers emerged earlier than male flowers. Although both flower types exhibited temporal fluctuations from anthesis to senescence, the relative abundance of hermaphroditic flowers declined progressively over time, whereas that of male flowers increased.

A significant association between inflorescence size and abundance (Wald χ^2^ = 239.57, *p* ≤ 0.001) was identified, with smaller inflorescences being relatively more abundant than large inflorescences (one-flowered: 44.12 ± 3.9%; six- and seven-flowered: 1.84 ± 1.5% and 1.25 ± 1.2%, respectively; Figure 3C). Moreover, the frequencies of both floral types were significantly affected by inflorescence size (hermaphroditic: Wald χ^2^ = 45.58, *p* ≤ 0.001; male: Wald χ^2^ = 47.48, *p* ≤ 0.001; Figure 3D). The frequency of hermaphroditic flowers decreased (r = −0.846, *p* ≤ 0.05) with increasing inflorescence size, whereas that of male flowers increased (r = 0.934, *p* ≤ 0.01). Male flowers consistently outnumbered hermaphroditic flowers across all inflorescence sizes.

### 2.3. Effects of Resource Limitation on Floral Sex Expression

**Figure 4:**
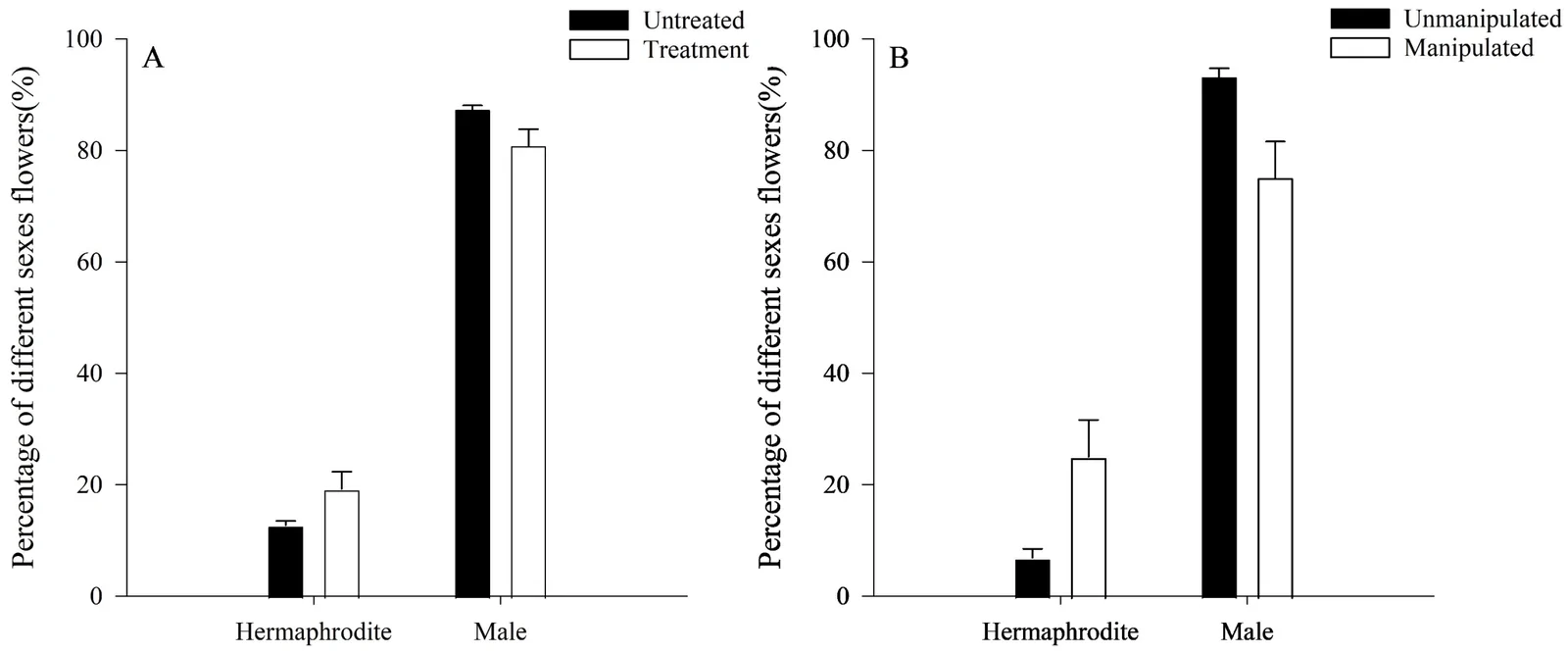
Percentage of hermaphroditic and male flowers of *Punica granatum* L. (**A**) in untreated and treated groups (*n* = 10 trees per treatment), and (**B**) flower sex percentages in unmanipulated and manipulated treatments (*n* = 120 labelled inflorescences per treatment) (mean ± SE).

Fertilization significantly affected the sex differentiation of flowers (Wald χ^2^ = 8.29, *p* ≤ 0.01) (Figure 4A). In the untreated (unfertilized) group, the frequency of hermaphroditic and male flowers was 12.72 ± 0.78% and 87.28 ± 0.78%, respectively. In the treatment group, the frequency of hermaphroditic and male flowers was 19.26 ± 3.06% and 80.74 ± 3.06%, respectively.

The proportion of hermaphroditic flowers was 6.87 ± 1.62% in the unmanipulated group and 25.00 ± 6.60% in the manipulated group. The probability of hermaphroditic flower occurrence was significantly higher in the manipulated group than in the unmanipulated group (Wald χ^2^ = 13.00, *p* ≤ 0.001) (Figure 4B). This indicates that the removal of supernumerary flower buds significantly increased the probability of the first flower bud developing into a hermaphroditic flower.

### 2.4. Pollen Production, Viability, and Pollen Removal Rate

**Figure 5:**
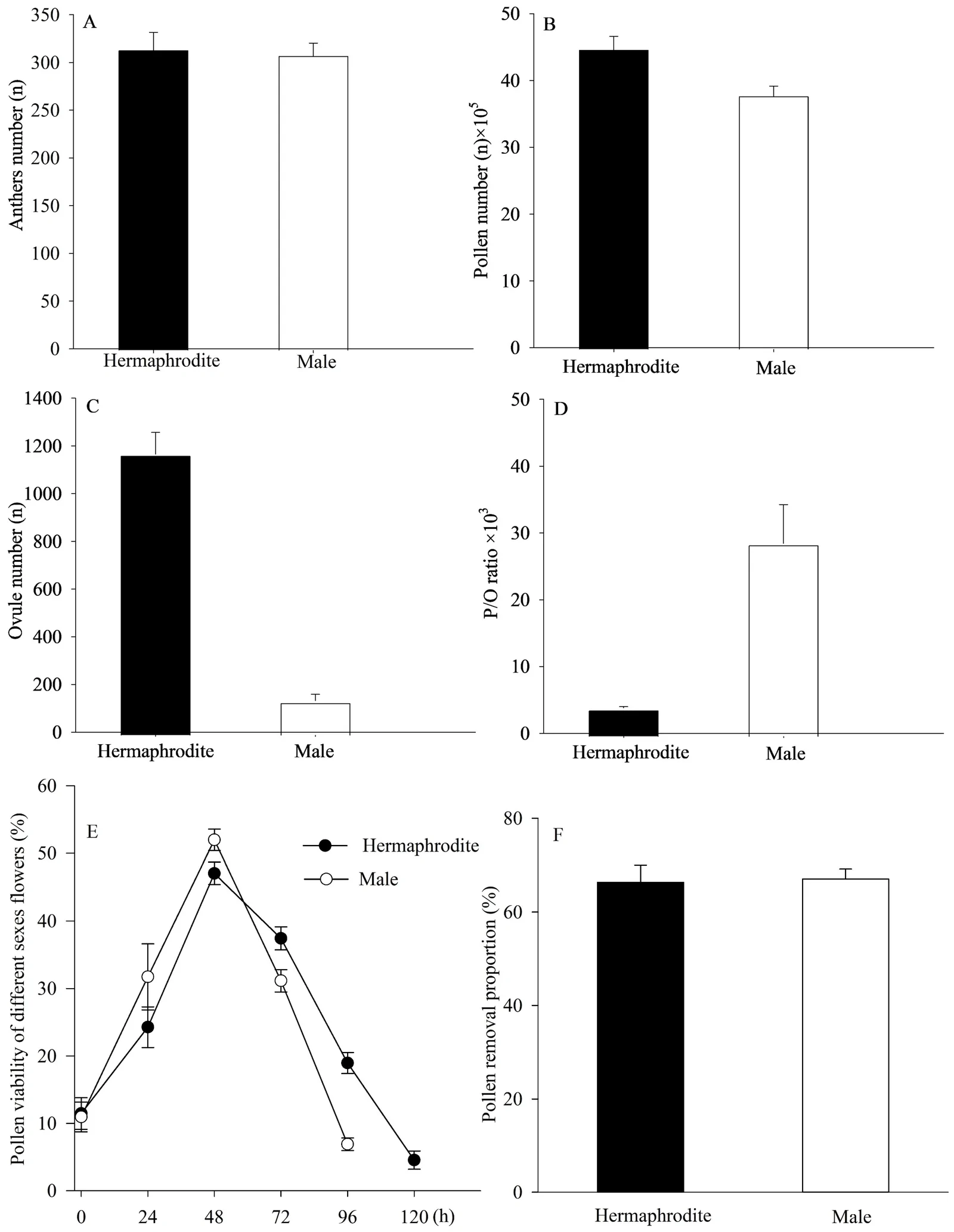
(**A**–**D**) Sex allocation characteristics (*n* = 15 flowers per flower-sex morph), (**E**) pollen viability (*n* = 20 flowers per flower-sex morph) and (**F**) pollen removal (*n* = 15 flowers per flower-sex morph) pollination characteristics of flowers of different sexes in *Punica granatum* L. (mean ± SE).

Significant differences in anthers number (Wald χ^2^ = 5.26, *p* ≤ 0.05), pollen production per flower (Wald χ^2^ = 8.02, *p* ≤ 0.05), ovule number (Wald χ^2^ = 210.95, *p* ≤ 0.001), and pollen/ovule (P/O) ratio (Wald χ^2^ = 18.07, *p* ≤ 0.001) were detected between the two floral types. Hermaphroditic flowers surpassed male flowers in terms of anther number (hermaphroditic: 312.90 ± 18.64, male: 306.90 ± 13.48), pollen count (hermaphroditic: 44.59 ± 2.02, male: 37.67 ± 1.50), and ovule number (hermaphroditic: 1165.50 ± 91.07, male: 132.40 ± 26.43), whereas the P/O ratio was higher in male flowers (28.45 ± 5.79) than in hermaphroditic flowers (3.83 ± 0.17) (Figure 5A–D). Pollen viability followed similar temporal trajectories in both floral types, peaking at 48 h, coincident with full anther dehiscence, and declining thereafter. Male flowers tended to show higher viability during the early phase (0–48 h), with the largest difference observed at 24 h, whereas hermaphroditic flowers retained relatively higher viability at 72 h and 96 h. By 120 h, pollen viability had dropped to minimal levels in hermaphroditic flowers (Figure 5E). The proportion of pollen removed during anthesis did not differ significantly between flower types (Wald χ^2^ = 0.03, *p* ≥ 0.05) (Figure 5F).

### 2.5. Floral Rewards and Visiting Frequency

**Figure 6:**
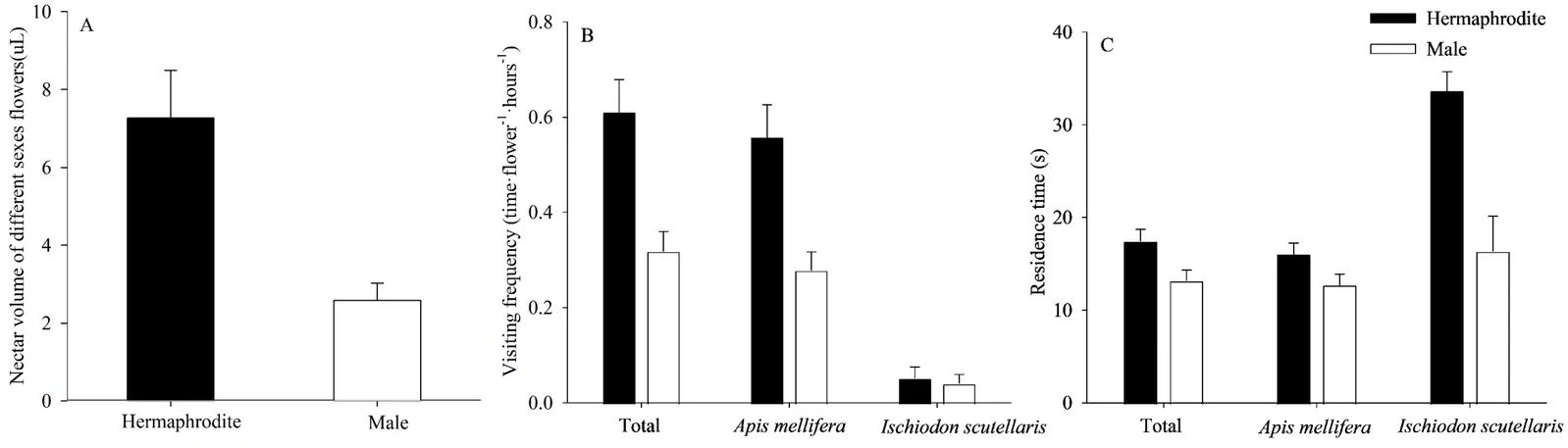
Pollination characteristics of *Punica granatum* L. flowers of different sexes, including (**A**) nectar volume, (**B**) visiting frequency of insect pollinators, and (**C**) visiting duration of insect pollinators (mean ± SE, *n* = 20 flowers per flower-sex morph).

Both floral types secreted nectar, with significant differences in volume (Wald χ^2^ = 14.87, *p* ≤ 0.05). Hermaphroditic flowers produced nearly three times more nectar (7.26 ± 1.23 μL) than male flowers (2.59 ± 0.44 μL) (Figure 6A). *Apis mellifera* and *Ischiodon scutellaris* carried pollen on their body surfaces and were therefore considered pollinators. The total visiting frequency and residence time differed significantly between the two floral types (Wald χ^2^ = 14.57, *p* ≤ 0.001; Wald χ^2^ = 6.37, *p* ≤ 0.05, respectively) (Figure 6B,C). Hermaphroditic flowers were visited by *A. mellifera* more frequently than male flowers (Wald χ^2^ = 14.55, *p* ≤ 0.01), whereas *I. scutellaris* showed no such preference for flower type (Wald χ^2^ = 0.13, *p* ≥ 0.05). Overall, *A. mellifera* exhibited a higher daily visitation rate than *I. scutellaris* (Figure 6B). Residence time also varied significantly between flower types for both insect species (*I. scutellaris*: Wald χ^2^ = 13.48, *p* ≤ 0.05; *A. mellifera*: Wald χ^2^ = 3.99, *p* ≤ 0.05) and was consistently longer on hermaphroditic flowers than on male flowers for both species. However, interspecific difference in residence time was significant only for hermaphroditic flowers (Wald χ^2^ = 18.59, *p* ≤ 0.001), not for male flowers (Wald χ^2^ = 1.20, *p* ≥ 0.05) (Figure 6C).

### 2.6. Reproductive Ability of the Two Flower Morphs

Fruit set differed significantly between flower morphs across pollination treatments (Table 2). As predicted from the morphological analysis (Section 2.1), male flowers had ovule structures, yet no fertile ovules were formed, thereby precluding fertilization and fruit set with seed development in any treatment and confirming their female sterility. Hermaphroditic flowers exhibited substantial variation in fruit set across treatments (Wald χ^2^ = 419.65, *p* ≤ 0.001). The highest fruit set percentage was recorded after supplementary hand pollination (83.26 ± 0.81%), whereas the lowest rate was detected after emasculation with natural pollination (43.60 ± 2.04%). Intermediate rates were recorded in the spontaneous autogamy (63.15 ± 0.83%), hermaphroditic × male cross (64.19 ± 1.65%), and natural pollination (50.88 ± 0.88%) groups. The reciprocal cross (male × hermaphroditic) produced no fruit. In contrast, seed number and seed set did not differ among treatments (Wald χ^2^ = 0.20, *p* ≥ 0.05).

## 3. Discussion

### 3.1. Sexual System Characterization of P. granatum

In Kashi city, southern Xinjiang, *P. granatum* plants developed hermaphroditic and male flowers, a pattern consistent with andromonoecy, as previously documented for this species [51,52]. We detected that the morphological divergence between the two types of flowers was primarily expressed in organ traits, with hermaphroditic flowers exhibiting significantly larger corolla, pistil, style, ovary, stamen-insertion base depth, and pollen grain surface area, whereas male flowers contained reduced or degenerated pistil organs and a style either equal to or shorter than the stamens (Figure 1; Table 1). Morphologically and histologically, the two morphs correspond to the flower types described by Wetzstein et al. [52]. The hermaphrodite (hermaphroditic) flowers possess a discoid, exudate-covered stigma, an elongate style, and numerous anatropous ovules, whereas the flowers we refer to as male are functionally male, retaining well-developed stamens but with pistils shortened to variable heights and ovules that are rudimentary and degenerate. These features were consistent across analyzed individuals, confirming a stable andromonoecious sexual system in this species, in agreement with earlier reports for other cultivars and production regions [51,56,57].

In andromonoecious taxa, hermaphroditic flowers typically surpass male flowers in terms of size and biomass. However, the magnitude of this divergence varies among species. For example, in *Soranthus meyeri* (Apiaceae), hermaphroditic flowers are larger and produce more pollen than male flowers, consistent with their higher resource investment [60]. In *Capparis spinosa* and *Veratrum nigrum*, pollen quantities do not differ between pollen types, despite their superior size and biomass [61,62]. In *Acer oblongum*, hermaphroditic flowers exhibit greater biomass and style length than either staminate type [63]. In our study, hermaphroditic flowers significantly exceeded male flowers in terms of morphological metrics, single-flower biomass, and female-organ biomass allocation (Figure 2; Table 1). In contrast, male flowers allocated proportionally more biomass to the petals, calyces, and stamens than hermaphroditic flowers (Figure 2B). These allocation patterns were accompanied by considerable variation in anther number, pollen number, ovule number, and P/O ratio (Figure 5A–D). In pomegranate, the ovules of hermaphroditic flowers are the structures that, upon fertilization, develop into the arils that make up the edible portion of the fruit, and aril number rather than aril size is the principal determinant of fruit size [64], whereas the ovules of male flowers are rudimentary and degenerate [52]. The greater ovule number recorded here in hermaphroditic flowers is therefore consistent with their female function and with the central role of ovule number in fruit development [53,64]. Hermaphroditic flowers produced more pollen and ovules, whereas male flowers exhibited a higher P/O ratio, a pattern consistent with resource optimization for male function. Overall, the observed gradients in morphological traits, biomass, and sex allocation from hermaphroditic to male flowers suggest that male flowers arise from pistil degeneration in certain hermaphroditic flowers [52,53,65], likely under resource constraints.

### 3.2. Floral Sex Expression Dynamics and Resource Dependence

Sex expression in plants can shift during development or in response to environmental conditions, reflecting a strategy to optimize fitness under variable resource availability [9,14]. Under resource-limiting conditions, increased allocation to male function and a male-biased sex ratio are frequently observed [66]. In andromonoecious species, the phenological patterns of hermaphroditic and male flowers vary widely. For example, in *V. nigrum* and *Leptospermum scoparium*, opening of hermaphrodite flowers precedes that of staminate flowers during the initial blooming phase [61,67], whereas in *Capparis spinosa* and *Solanum carolinense*, male flowers dominate the early blooming phase [29,62]. Moreover, in *A. oblongum*, staminate type I flowers bloom first, followed by hermaphroditic and staminate type II flowers [63]. Martínez et al. investigated floral morphological variation among three pomegranate clones, revealing that putative “male” flowers are hermaphroditic flowers with atrophied pistils and that the ratio of flower sex types varies significantly among genotypes and years [55]. Comparable genotype-, season-, and position-dependent variation in the ratio of flower types has since been reported in other pomegranate-growing regions [54,56,57]. Within a flowering wave, floral quality also depends on flower position and size. Single and terminal flowers are larger and contain more ovules than lateral flowers, and lateral flowers are frequently affected by ovule degeneration severe enough to reduce fruit set [53].

*Punica* spp. are mainly distributed in subtropical and temperate regions, where the natural environment affects plant growth and reproduction. In the present study, hermaphroditic flowers bloomed earlier than male flowers at the floral, individual, and population levels. Over the flowering season, the proportion of hermaphroditic flowers declined progressively, whereas the relative abundance of male flowers increased (Figure 3B). Similarly, the frequency of hermaphroditic flowers decreased with increasing inflorescence size, whereas that of male flowers increased (Figure 3C,D). This positional gradient is consistent with the inflorescence-level differences in floral quality documented by Wetzstein et al. [53] and with the observation that single hermaphrodite flowers occur more frequently than terminal or lateral hermaphrodite flowers within a cluster [56]. This temporal and spatial shift from the female to male system, along with the consistently higher proportion of male flowers compared with hermaphroditic flowers across all levels, supports the idea that the male-biased sexual system of this species grows under resource limitations. A male-biased flower ratio is characteristic of the species, with male flowers commonly outnumbering hermaphroditic flowers [54,56,57].

Our resource manipulation experiments provide support for this interpretation. Fertilized plants produced significantly more hermaphroditic flowers and fewer male flowers than control plants, whereas bud removal from inflorescences enhanced the frequency of the first flower developing into a hermaphroditic form. These findings provide evidence that increased resource supply is associated with increased production of hermaphroditic flowers, whereas reduced resource input is associated with a male-biased sex ratio. Although soil or leaf nutrient concentrations were not measured, the results are consistent with the hypothesis that resource availability influences sex determination in this species. Growth-regulator applications similarly shift the proportion of flower types toward male flowers in this species, indicating that sex expression is plastic and hormonally responsive [56]. The effects of nutrient availability on floral sex ratio have been previously demonstrated under controlled conditions [37]. Our study extends these findings to an orchard setting, providing field-scale evidence consistent with resource-associated sex allocation in mature pomegranate trees under production conditions, in line with the optimal resource reallocation hypothesis [23]. We propose that the production of male flowers may represent a resource-saving allocation strategy under constrained environmental conditions, consistent with the hypothesis that male flowers are resource-saving artifacts rather than functional reproductive structures.

### 3.3. Pollination Biology, Mating Patterns, and Reproductive Assurance

Plant mating systems include selfing, outcrossing, and mixed mating, with the latter being common in nature [68,69,70,71]. Four insect species visited pomegranate flowers in the orchard: *Tetramorium* sp., Syrphid fly sp., *Apis mellifera*, and *Ischiodon scutellaris*. Microscopic examination showed that pomegranate pollen grains were present on the body surfaces of *A. mellifera* and *I. scutellaris* (Figure 1), and so we identified these species as pollinators. Syrphid fly sp. and *Tetramorium* sp. only visited the inflorescences before anthesis to feed on the exudate secreted at the tip of floral buds and were not considered pollinators. Hermaphroditic flowers, with longer longevity (5.6 vs. 4.6 d) and threefold higher nectar content, were associated with greater visitation, longer residence, and more pollen removal than male flowers. *A. mellifera* exhibited a clear preference for hermaphroditic flowers (Figure 6). Controlled crosses confirmed the female sterility of male flowers (no fruit set when male flowers served as maternal parents), whereas the fruit set of hermaphroditic flowers varied significantly across treatments. The reciprocal cross, using male flowers as female parents, failed. Histological evidence is consistent with this complete pistil sterility. Although the stigmatic papillae of male flowers support pollen germination, pollen tubes are rarely observed within their styles, and their ovules are rudimentary and degenerating [52]. Seed number and set did not differ among treatments. A higher rate of fruit set under autogamous conditions compared to that under natural conditions suggests reproductive assurance, whereas reduced fruit set under natural conditions compared to that achieved through hand pollination indicates pollen limitation. This pattern is consistent with the arid study environment. Collectively, these results indicate that male flowers are reduced structures that retain a direct paternal function through viable pollen. Meanwhile, the increased autogamous rather than natural fruit set suggests that hermaphroditic flowers achieve reproductive assurance when pollinator services are unreliable.

### 3.4. Ecological and Evolutionary Implications

These findings provide valuable insights into the evolution of andromonoecy and maintenance of male flowers in flowering plants. Our results are consistent with the optimal resource reallocation hypothesis [23] as a plausible explanation for the maintenance of andromonoecy in this species, while not excluding the contributory role of pollen donation. Nevertheless, the strong cultivar-, season-, and position-dependent variation in flower-type ratios reported for this species [53,54,55,56,57] indicates that the strength of resource-driven sex expression should be evaluated across environments and genotypes. Male flowers secreted less nectar and received fewer pollinator visits than hermaphroditic flowers, suggesting that their contribution to pollinator attraction was limited. Instead, the primary selective advantage of male flowers may be resource saving, which allows plants to allocate limited resources to fewer but more successful hermaphroditic flowers. However, we acknowledge that no manipulative experiment (e.g., removal of male flowers) was conducted to directly test the pollinator-attraction function. This hypothesis requires further experimental validation. The observed association between resource supply and sex expression, combined with the complete female sterility of male flowers, suggests that these structures are associated with resource limitation and the phenotypic outcomes of resource competition among developing flower buds, rather than being organs specifically evolved for pollen donation. As only hermaphroditic flowers set fruit, and because fruit size in pomegranate is determined chiefly by the number of fertilized ovules rather than by aril size [53,64], resources invested in male flowers that never set fruit are directly relevant to orchard productivity. Genotypes or management practices that shift the ratio towards hermaphroditic flowers are therefore expected to favor yield [53,56,57].

This interpretation is supported by the temporal dynamics of sex expression. The proportion of male flowers increases as the flowering season progresses, possibly as a consequence of progressive resource depletion, although this hypothesis was not tested in the present study. Similarly, in larger inflorescences, where resource competition among flowers is relatively intense, male flowers dominate. These patterns support the association of male flower development with resource limitation, rather than an adaptive strategy facilitating pollen dispersal.

Male flowers achieved higher peak pollen viability than hermaphroditic flowers, although pollen viability declined more rapidly over time. Combined with female sterility, this suggests that pistil degeneration in male flowers does not fully block the development of pollen capable of high initial viability. However, pollen longevity is still modulated by floral developmental programming. The pollen of the two flower types is similar in size and in vitro germination [52], although viability and germination can differ between the two morphs depending on cultivar [72]. This observation warrants further investigation into the genetic or hormonal controls underlying pistil abortion and pollen persistence. Understanding the genetic basis of pistil degeneration in this system may provide insights into the developmental plasticity of floral sex expression and its evolutionary consequences.

## 4. Materials and Methods

### 4.1. Material and Study Site

*P. granatum* is globally distributed and has a long history of cultivation. In China, more than 280 cultivated varieties (types) have been reported [73]. This species produces radially symmetrical flowers with a vase-, tube-, or bell-shaped morphology. The flowers, characterized by bright red petals and an equal number of sepals, can be solitary or aggregated in racemes. The leaves are arranged either oppositely or in clusters. Flowering occurs from May to July, whereas the fruiting period spans September to October. The fruit develops from the inferior ovary and exhibits a spherical or oblate form. During winter, plants enter a dormant state and develop cold tolerance. Pomegranate plants require sufficient light, are drought-resistant and are typically distributed in areas with annual precipitation ranging from 55 to 1600 mm [73]. In Xinjiang, extended periods of low temperatures during winter have promoted the adoption of the creeping cultivation method in which plants are buried under soil for overwintering [73,74].

Kashi city is located in Xinjiang and is a special economic zone in China. It has a temperate desert climate characterized by abundant sunshine and limited precipitation (average annual precipitation of 70 mm), with an annual average temperature of 12 °C and 2800 h of sunshine. In this study, we focused on the pomegranate orchards in Awati Township, Kashi City (39°28′58″ N, 76°9′27″ E; altitude: 1280 m). Plant material consisted of local sweet-flavored Kashi pomegranate germplasm, a traditional landrace group widely cultivated in Kashi without formal cultivar registration. The study was carried out in a 1000 m^2^ orchard. We selected 20 healthy representative trees for sampling. Trees were 25 years old and planted at a spacing of 3 m. Trees were managed under local conventional orchard practices: flood irrigation was applied and fertilization followed local orchard standard regimes.

From 2019 to 2021, investigations were conducted in these orchards to comprehensively examine the sexual systems of pomegranate. No field experiments were performed in 2020 owing to COVID-19-related restrictions. Pollination and pollinator-related experiments were conducted between 2019 and 2021. Most morphological measurements were completed in 2019, and supplementary sampling was conducted in 2021.

### 4.2. Floral Morphology, Biomass, and Resource Investment

Individuals of this species produce two types of flowers (vase- and bell-shaped). From early to mid-June, when orchard trees were in full bloom, 15 fresh flowers per floral morph were collected. Three flowers were sampled from solitary inflorescences at the same position on each of five individual trees. We measured the corolla diameter; length and width of corolla tube, petals, calyces, anthers, and ovary; length of style, stamens, and filaments; stamen distribution; and distance from stamen base using SE 2000 electronic calipers (accuracy of 0.02 mm; Holy Instrument and Technology Co., Ltd., Guilin, China). Style length was defined as the vertical distance from the stigma base to the top of the ovary. Ovarian length refers to the vertical distance from the top of the ovary to the bottom of the flower receptacle. The anthers of the two types of flowers were placed in 2 mL Eppendorf tubes, allowed to dry naturally for 3 days, pasted on the specimen table using electric glue (5 mm blue core), and coated with a carbon film using a vacuum coating instrument (MSP-2S, IXRF Systems, Inc. Austin, TX, USA). The pollen grains were observed and photographed using a scanning electron microscope (FEI, Quanta 250, FEI Company, Hillsboro, OR, USA). Stamen insertion base depth (mm) was the vertical distance from the insertion point of the stamens down to the base of the flower receptacle. The surface area of the pollen grains was determined under a microscope.

Fifteen newly opened flowers with indehiscent anthers were collected from each type of flower and immediately transported to the laboratory. Ten pollen grains were measured from each of the three anthers of every flower. The fresh biomass of the whole flower and its individual parts (petal, calyx, and stamen) was measured using an FA1004 electronic balance (accuracy of 0.0001 g; Minqiao Precision Scientific Instrument Co., Ltd., Shanghai, China). These measurements were used to compare resource allocation among flower types and to test whether male flowers represented a reduced investment morphology.

### 4.3. Sexual System and Floral Sexual Expression

Before the flowering period, we pre-tagged 20 floral buds of each sex at comparable canopy positions on solitary inflorescences from 20 trees. Flower sex was determined at the bud stage based on external bud shape. Hermaphrodite flowers exhibited urn-shaped buds, whereas male flowers showed campanulate (bell-shaped) buds. We recorded flower morphology, color, and lifespan. During the peak flowering period, we marked 20 trees and determined the number of flowers of each sex per week. We recorded the frequency distribution of inflorescence size per individual and the occurrence of each sex within the inflorescences. These observations enabled the characterization of the sexual system at the floral, inflorescence, individual, and population levels and facilitated the documentation of the temporal dynamics of sex expression.

### 4.4. Effects of Resource Limitation on Floral Sex Expression

To determine whether resource limitation influenced floral sex expression, particularly the production of male and hermaphroditic flowers, we conducted two complementary experiments. First, 20 plants of comparable size were selected from the orchard. The experiment was arranged in a randomized design with two subgroups: 10 trees were assigned to the treatment group and 10 trees to the untreated group. Starting in mid-May (early bloom stage) and continuing until late June (end of flowering stage), foliar sprays were applied to the tree canopy of the treatment group every two weeks. The spray solutions were prepared at the concentrations recommended by the manufacturers: fluid boron (trace-element water-soluble fertilizer, Naifei Agricultural Science and Technology Development (Shandong) Co., Ltd., Weifang, China) was diluted 1500-fold, a flight-control phosphorus–potassium fertilizer (macro-element water-soluble fertilizer, Naifei Agricultural Science and Technology Development (Shandong) Co., Ltd.) was diluted 1000-fold, and a 13-element macro-element water-soluble fertilizer (Boruisite Agricultural Science and Technology Development (Shandong) Co., Ltd., Weifang, China) was diluted 1500-fold. Foliar sprays were applied to the tree canopy using a handheld sprayer until the leaves were uniformly wetted. During the young bud stage, the canopy was sprayed with a mixture of flight-control phosphorus-potassium fertilizer and 13-element water-soluble fertilizer to supply macroelements and trace elements to support bud growth. When entering the initial anthesis and full bloom stages, the spray formula was switched to a mixture of flight-control phosphorus-potassium fertilizer and fluid boron. This formulation was used to promote pollen germination, pollen tube elongation, and maintain floral vitality. The untreated group received regular irrigation without additional foliar fertilizer application. No soil or leaf nutrient analyses were performed to verify the fertilization effect. During the flowering period, the number of male and hermaphroditic flowers per plant was recorded. Second, from untreated plants, a cohort of inflorescences with newly formed flower buds of uniform size was selected and subjected to the following treatments: (i) unmanipulated (no bud removal) and (ii) manipulated retention of the first flower bud and removal of the other two buds. The sex expression patterns of the flowers on each inflorescence were recorded.

### 4.5. Pollen Production, Viability, and Pollen Removal Rate of Both Sexual Type Flowers

#### 4.5.1. Pollen Production and Pollen/Ovule Ratio

Following the method described by Dafni et al. [75], 15 flowers of each type were selected from the same position on the inflorescence derived from 15 different individuals, and the number of anthers per flower was counted. We randomly collected 10 anthers from each flower and placed them in separate 2 mL microcentrifuge tubes. The anthers were thoroughly crushed, and 2 mL of water was added, followed by shaking to obtain a pollen suspension; 5 µL of the pollen suspension was placed onto a glass slide five times using a micro-sampler (accuracy 5 µL; Gaoge Industry & Trade Co., Ltd., Shanghai, China). Each flower was sampled in three independent replicates, and pollen counts were determined using a Motic B1 optical microscope (Motic China Group Co., Ltd., Xiamen, China). Simultaneously, pollen grain length was measured along the polar (P) and equatorial axes (E) using a optical microscope (ECLIPSE Ci-L, Nikon Corporation, Tokyo, Japan). Subsequently, the pollen surface area (projected two-dimensional area of pollen grains) was calculated using the formula reported by Zhang and Tan [76]: S = (π × P × E)/4. Following the method described by Dafni et al. [75], the ovaries of the two flower types were dissected using anatomical needles, and the number of ovules per flower was recorded to determine the P/O ratio.

#### 4.5.2. Pollen Viability

Pollen viability was determined using the 2,5-diphenyl monotetrazolium bromide (MTT) method [75]. Before flowering, we selected 20 flowers of each type at the same developmental stage from 20 different individuals and covered them with white nylon mesh bags. At different stages of flowering (from newly blossoming flowers with indehiscent anthers to the end of florescence), fresh pollen was collected every other day. These pollen grains were placed on glass slides and stained with one to two drops of MTT solution to analyze viability. Heat-killed pollen grains were used as controls. The total number of pollen grains and viable pollen grains was counted using a Motic B1 optical microscope (Motic China Group Co. Ltd., Xiamen, China). According to the method established by Dafni et al. [75], viable pollen was stained (purple), whereas inactive pollen remained colorless.

#### 4.5.3. Pollen Removal During Anthesis

During the flowering period, we tagged 20 flowers of each sex at the same developmental stage (before anthesis) from 20 different individuals on different single-flower inflorescences. When petal abscission began, the tagged flowers were transported to the laboratory. During anthesis, pollen removal per flower was quantified under an optical microscope following the method of Abdusalam et al. [77]. For each tagged flower, pollen grains were counted twice: at flower opening (initial pollen number) and when petal abscission began (residual pollen number). Pollen removal per flower was calculated as the difference between the initial and residual pollen numbers, and the pollen removal proportion was calculated as pollen removal divided by the initial pollen number of the same flower.

### 4.6. Nectar and Pollinator Visitation

Twenty flowers of each sex were selected from single inflorescences located at the same positions in 20 different individuals [75]. The flowers were allowed to blossom and were marked using white nylon mesh bags. Nectar secretion was measured using a capillary tube (0.9–1.1 mm inner diameter × 100 mm length; Jiyu Experimental Equipment Co., Ltd., Shanghai, China) every other day from the start to the end of the flowering stage. Nectar volume was measured following the method of Zhang and Tan [76]. The visiting frequency, visiting duration, and behavior of the pollinator insects were observed for each flower type. Three or four of these insects were captured using insect nets for taxonomic identification. Pollen grains that adhered to the insect body surface were brushed onto glass slides and identified under a light microscope. Insects that were found to be carrying pomegranate pollen were identified as the main flower-visiting insect species. Bagging experiments showed that flowers excluded from insect visits failed to set fruit, whereas open pollinated flowers produced fruits. This result indicated that insect visitors as a whole were indispensable for fruit production in the orchard. These pollen-carrying floral visitors likely function as pollinators. On a sunny day, 12 flowers of each type that opened on the same day in a single inflorescence were randomly tagged. Pollinator visitation frequency and duration were recorded daily from 09:30 to 17:30 over four to six consecutive days during anthesis. Each flower type was observed for 15 min every hour (120 min of observation per day).

### 4.7. Controlled Pollination Experiments

During the full bloom stage, 20 flowers at the pre-anthesis stage with good developmental status on 20 different individual plants in the orchard (at positions consistent with single-flower inflorescences) were selected and tagged for each treatment. During opening, the flowers of each sex type were subjected to the following treatments: (1) natural pollination (no emasculation, no bagging), (2) spontaneous autogamy (no emasculation, with net bagging), (3) hand pollination after natural pollination (no emasculation, artificial addition of conspecific pollen), (4) natural pollination with anther removal (emasculation, no bagging), (5) hermaphroditic × male flower crosses, and (6) male × hermaphroditic crosses. When the ovary expanded in these inflorescences, the net bags were removed, and the fruit and seed sets were compared when the fruits matured [77]. These experiments were designed to investigate whether male flowers contribute to reproduction through pollen donation or female function.

### 4.8. Data Analysis

All data were analyzed using SPSS 23.0. Normality and homogeneity of variance were evaluated before subsequent analysis. Differences among flower types in floral morphological traits, flower biomass, anther number, ovule number, pollen number, P/O ratio, flower longevity, and occurrence frequency were analyzed using generalized linear models (GLMs) without incorporating tree identity as a random effect. As pollen viability was measured only to characterize its temporal dynamics, these data were not included in the statistical analyses and are presented descriptively in Figure 5E. For the variables analyzed using GLMs, the distribution family and link function were selected according to the nature of each response variable. The nectar amount (continuous variable) was analyzed using a Gaussian distribution with an identity link function. The pollinator visitation frequency (count data) was analyzed using a Poisson distribution with a log link function. Fruit- and seed-setting rates (binomial proportion data) under different pollination treatments were analyzed using a binomial distribution with a logit link function. Floral-sex proportions under different resource limitation treatments were analyzed using a binomial distribution with a logit link function. Pearson’s correlation analysis was used to examine the relationships between inflorescence size and the frequencies of hermaphroditic and male flowers. Statistical data are expressed as mean ± standard error (mean ± SE). All figures were prepared using SigmaPlot 10.0.

## 5. Conclusions

The results of this study are consistent with a typical andromonoecious sexual system in *P. granatum* in which male flowers arise from pistil degeneration of hermaphroditic flowers, possibly in response to resource limitations. These male flowers are morphologically reduced, with minimal investment in female reproductive structures. They exhibit female sterility and cannot set fruit, yet they produce viable pollen capable of siring progeny. Analyses of the spatial and temporal patterns of sex expression, together with resource-manipulation experiments, provide evidence that increased resource supply is associated with increased production of hermaphroditic flowers, a pattern consistent with the optimal resource-reallocation hypothesis [23]. Thus, male flowers may represent a resource-saving response to resource constraints. Whether male-flower proportion can serve as a reliable indicator of plant resource status or environmental stress, including its potential application in orchard management to guide fertilization regimes, remains to be validated across different environments and genotypes. From a functional perspective, the viable pollen produced by male flowers contributes to pollination, indicating that male flowers should not necessarily be regarded as a complete yield loss. These findings advance our understanding of andromonoecy in cultivated fruit trees and provide a basis for further research on pomegranate reproductive biology and cultivation. One limitation of this study is that flowers collected from the same tree are not statistically independent, as they share the same microenvironment and resource supply. We used GLMs without a random term for tree identity, which may have affected the statistical inference. Future studies could employ GLMMs to account for clustered sampling.

## Figures and Tables

**Table 1:** Differences in floral characteristics between the two types of *Punica granatum* flowers with different shapes (mean ± SE, *n* = 15 flowers per flower sex morph).

Characteristic		Hermaphrodite	Male	Wald χ^2^_df=1_	*p* Value
Corolla tube	Length (mm)	29.05 ± 1.01	23.03 ± 0.44	44.07	*p* ≤ 0.001
	Width (mm)	16.54 ± 0.53	15.63 ± 0.41	NS	*p* ≥ 0.05
Corolla	Diameter (mm)	41.98 ± 1.42	34.38 ± 1.10	17.95	*p* ≤ 0.001
Sepal	Length (mm)	11.45 ± 0.16	10.88 ± 0.13	6.79	*p* ≤ 0.01
	Width (mm)	6.98 ± 0.13	6.77 ± 0.15	NS	*p* ≥ 0.05
Petals	Length (mm)	23.80 ± 0.52	22.90 ± 0.33	NS	*p* ≥ 0.05
	Width (mm)	18.86 ± 0.30	17.81 ± 0.25	6.60	*p* ≤ 0.01
Style	Length (mm)	17.74 ± 0.62	8.98 ± 0.54	106.49	*p* ≤ 0.001
Stamens	Length (mm)	6.17 ± 0.09	6.07 ± 0.08	NS	*p* ≥ 0.05
Ovary	Length (mm)	12.37 ± 0.73	6.52 ± 0.40	64.15	*p* ≤ 0.001
	Width (mm)	8.69 ± 0.39	5.06 ± 0.28	60.51	*p* ≤ 0.001
Filaments	Length (mm)	4.80 ± 0.11	4.88 ± 0.07	NS	*p* ≥ 0.05
Anthers	Length (mm)	2.11 ± 0.03	2.02 ± 0.02	6.11	*p* ≤ 0.01
	Width (mm)	1.70 ± 0.02	1.66 ± 0.02	NS	*p* ≥ 0.05
Stamen whorl number		6.11 ± 0.2	5.7 ± 0.3	NS	*p* ≥ 0.05
Stamen distribution (mm)		10.33 ± 0.36	8.95 ± 0.26	10.58	*p* ≤ 0.01
Stamen insertion base depth (mm)		5.48 ± 0.32	3.70 ± 0.12	41.98	*p* ≤ 0.001
Pollen grain surface area (μm^2^)		429.11 ± 23.65	349.52 ± 10.04	13.71	*p* ≤ 0.001

**Table 2:** Fruit and seed setting rates under different treatment conditions (mean ± SE, *n* = 20 flowers per flower sex morph).

	Treatment	Fruit Set (%)	No. Seeds per Fruit (n)	Seed Set (%)
Hermaphrodite	Natural pollination	50.88 ± 0.88	475 ± 39.94	40.76 ± 3.43
	Spontaneous autogamy	63.15 ± 0.83	476.83 ± 41.73	40.91 ± 3.58
	Hand pollination after natural pollination	83.26 ± 0.81	482.29 ± 56.28	41.38 ± 4.83
	Natural pollination after emasculation	43.60 ± 2.04	450.2 ± 44.07	38.63 ± 3.78
	Hermaphrodite × Male	64.19 ± 1.65	469.43 ± 37.71	40.28 ± 3.24
Male	Natural pollination	0	0	0
	Spontaneous autogamy	0	0	0
	Hand pollination after natural pollination	0	0	0
	Natural pollination after emasculation	0	0	0
	Male × Hermaphrodite	0	0	0
*p* value	*p* value	≤0.001	≥0.05	≥0.05

## Data Availability

The data presented in this study are available on request from the corresponding author.
